# Progesterone Primed Ovarian Stimulation (PPOS) *vs*. clomiphene Primed Ovarian Stimulation (CPOS) in high responder (HR) patients undergoing controlled ovarian stimulation. A Randomised Control trial

**DOI:** 10.5935/1518-0557.20240083

**Published:** 2025

**Authors:** Krishna Mantravadi Chaitanya, Durga Gedela Rao, Isha Gambhir

**Affiliations:** 1 Department of Embryology, Oasis India. Banjara Hills Road No 2, Hyderabad - 500034, India; 2 Department of Reproductive Endocrinology, Oasis India, Banjara Hills Road No 2, Hyderabad - 500034, India

**Keywords:** controlled ovarian stimulation, progesterone primed ovarian stimulation, clomiphene primed ovarian stimulation, IVF, LH surge

## Abstract

**Objective:**

To compare the efficacy and safety of PPOS and CPOS in high-responder patients undergoing COS for IVF.

**Methods:**

This one-year prospective, randomized, controlled trial included 86 high-responder patients. They were divided into PPOS (n=44) and CPOS (n=42). Both groups underwent COS with hormonal injections, and various parameters, such as LH surge, cycle cancellation rates, birth rates, implantation rates, and more, were measured and compared.

**Results:**

The study revealed that LH surge occurred in 2.3% of the PPOS group and 2.5% of the CPOS group, with no significant difference (*p*=0.9). The cycle cancellation rates were 9.1% for PPOS and 10% for CPOS. Birth rates were 57% for PPOS and 54% for CPOS. Implantation rates were 45% for PPOS and 49% for CPOS. There was no significant difference in the duration of stimulation (PPOS: 11.30±1.96 days, CPOS: 11.41±2.02 days, *p*=0.807) or the total FSH used (PPOS: 2888.95±791.80IU, CPOS: 2808±834.52IU, *p*=0.655). The PPOS group had a mean of 19.58±8.07 retrieved oocytes, while the CPOS group had a mean of 21.87±10.02, showing no significant difference (*p*=0.807). Similarly, there was no significant difference (*p*=0.376) in the number of mature (MII) oocytes between the PPOS group (15.67±6.23) and the CPOS group (17.08±7.96). Post-trigger LH levels were significantly lower in the PPOS group (PPOS: 49.68±27.54IU/L, CPOS: 71.83±43.43IU/L, *p*-value 0.007), indicating LH surge suppression. Neither group reported cases of ovarian hyperstimulation syndrome (OHSS).

**Conclusions:**

PPOS and CPOS offer similar outcomes in high-responder individuals undergoing COS for IVF, except for lower post-trigger LH levels in the PPOS group. Importantly, neither group experienced ovarian hyperstimulation syndrome (OHSS).

## INTRODUCTION

One of the most common infertility treatments is controlled ovarian stimulation (COS). COS involves the use of gonadotropins, which are hormones that help to stimulate the ovaries to produce multiple eggs. Once multiple eggs have been produced, they can be retrieved and fertilized in vitro (Howie & Kay, 2018).

During controlled ovarian stimulation (COS) with gonadotropins, the ovarian follicular response can vary significantly among patients and even within the same patient from cycle to cycle. An effective follicular response is crucial for successful assisted reproductive technology (ART) treatment. Patients are categorized as poor, normal, or high responders based on their response to ovarian stimulation. Increased follicles and oocytes characterize a high ovarian response to conventional ovarian stimulation (OS) compared with a normal response. High responders have an increased risk of ovarian hyperstimulation syndrome (OHSS) (Jirge, 2016; Prodromidou *et al.*, 2021).

A premature LH (luteinizing hormone) surge, marked by an early and unanticipated elevation in LH levels during the menstrual cycle, can occur prior to the full maturation of ovarian follicles. Healthcare providers often implement strategies involving GnRH agonists or antagonists to manage LH surges effectively (Shrestha *et al.*, 2015; Gallos *et al.*, 2017).

Clomiphene Primed Ovarian Stimulation (CPOS) employs clomiphene citrate to stimulate ovulation by promoting gonadotropin release, ovarian follicle development, and corpus luteum function. Clomiphene selectively binds to estrogen receptors, acting as both an estrogen agonist and antagonist, particularly in the hypothalamus, where it enhances negative feedback inhibition and increases gonadotropin secretion, including luteinizing hormone (LH), follicle-stimulating hormone (FSH), and testosterone (Diamond *et al.*, 2015; Mbi Feh *et al.*, 2024). CPOS increases the number of eggs produced by blocking estrogen effects on the ovaries, with the choice of protocol depending on factors like age, ovarian reserve, and risk of ovarian hyperstimulation syndrome (OHSS) (Kamath *et al.*, 2017). However, no studies regarding CPOS in high responders have been found.

Progesterone inhibits the LH (luteinizing hormone) peak during the menstrual cycle primarily by exerting negative feedback on the hypothalamus and pituitary gland. It reduces the frequency of GnRH (gonadotropin-releasing hormone) pulses released by the hypothalamus, decreasing the frequency of LH pulses. Additionally, progesterone suppresses the amplitude of LH pulses, making them less intense. These combined effects of progesterone help prevent further ovulation after the initial LH surge that triggers ovulation. This inhibition is crucial for maintaining the stability of the luteal phase of the menstrual cycle and creating a conducive uterine environment for potential embryo implantation and pregnancy. If fertilization does not occur, declining progesterone levels lead to the breakdown of the corpus luteum, menstruation, and the start of a new menstrual cycle (DeMayo *et al.*, 2002; Guan *et al.*, 2021). PPOS is generally considered a safer option for women at high risk of OHSS. PPOS may also be less costly and time-consuming than traditional COS (Guan *et al.*, 2021).

In the context of high responders, the reviewed studies comparing Progesterone Primed Ovarian Stimulation (PPOS) with Clomiphene Primed Ovarian Stimulation (CPOS) during controlled ovarian stimulation offer pertinent insights. It has been suggested that elevated progesterone levels might not exert a significant clinical influence on treatment outcomes in high-responder patients (Requena *et al.*, 2014). A randomized controlled trial concluded that there is no clear advantage of either protocol, PPOS or CPOS, in various key outcomes for this specific patient group (Gambhir *et al.*, 2022). While adding clomiphene citrate to PPOS may modify some physiological responses, its impact on clinical outcomes in high responders is limited (Liu *et al.*, 2018). A study investigating the use of clomiphene citrate in combination with gonadotropin for preventing LH surges has shown promise. However, the study primarily involves fertile donors, which raises questions about its applicability to high responders in the broader context of assisted reproductive technology (Bhandari *et al.*, 2016).

These studies offer valuable insights, but more extensive research is required to determine the most effective protocol for controlled ovarian stimulation during in vitro fertilization, particularly focusing on high-responder patients. Currently, studies are scarce directly comparing PPOS and CPOS in high responders. Consequently, this research assessed the effectiveness and safety of PPOS versus clomiphene-primed ovarian stimulation (CPOS), specifically in high-responder patients undergoing controlled ovarian stimulation for IVF.

## MATERIALS AND METHODS

### Study setting

This one-year prospective, randomized, controlled trial was conducted at the Oasis Fertility Centre in Hyderabad, India.

### Study participants

**Figure 1:**
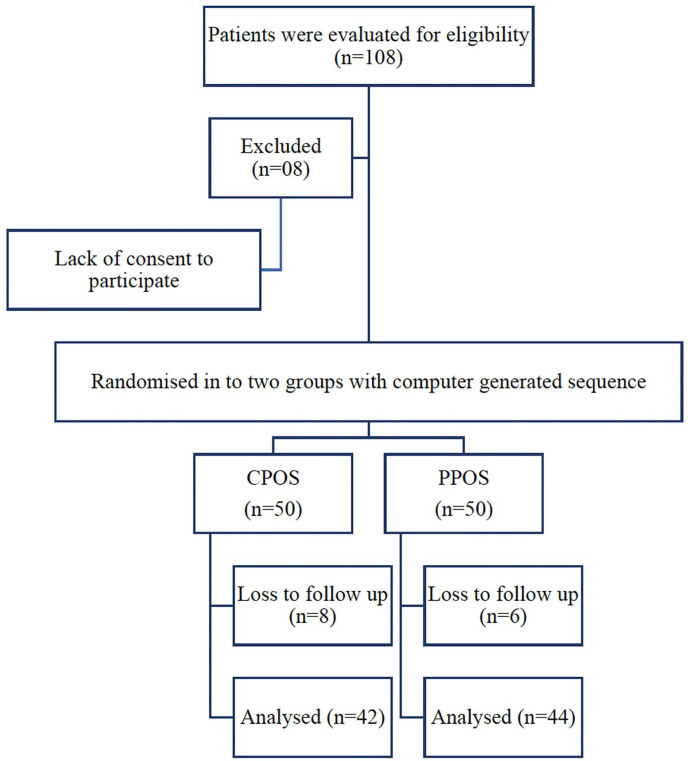
Consort diagram.

Patients between the ages of 18 and 35 years who were undergoing assisted reproductive technology (ART) procedures, such as in vitro fertilization (IVF) or intracytoplasmic sperm injection (ICSI), were screened for the high responder event. High responder patients are those with a history of excessive ovarian response (24 oocytes) during controlled ovarian stimulation and Antral follicle count (AFC) 24; antimullerian hormone (AMH) levels >3.4ng/mL Women over 35 years with a history of total fertilization failure, inadequate post-trigger hormonal levels, and incomplete oocyte retrieval were excluded from the study. Figure 1 (Consort diagram) below is a pictorial representation of the randomization of patient population into PPOs and CPOS arms respectively.

### Randomization

Using a computer-generated randomization list, 100 patients were assigned to either the progesterone-primed ovarian stimulation (PPOS) or the clomiphene-primed ovarian stimulation (CPOS) group. Fifty participants were enrolled in each group.

### Study groups

**-CPOS group:** Patients randomized into this arm received 150 mg/day of clomiphene citrate in divided doses of 50 mg 3 times per day starting from the eighth day of the cycle or when a leading follicle reached 14 mm in diameter, which was continued until the day of hCG injection.

**-PPOS group:** Patients randomized to this arm were administered Tab Medroxy Progesterone Acetate 10 mg/ day beginning on day 2 of the cycle in conjunction with the gonadotropins used for COS and continuing until the day of hCG injection.

### Procedure

Participants received gonadotropins from day 2 to day 14 of the menstrual cycle in both study arms. The initial dosage of gonadotropins was adjusted based on the participant’s body mass index (BMI), and subsequent changes were made in increments of 75IU.

### Follow-up

Monitoring of follicular growth was performed through transvaginal ultrasound examinations. Once the leading follicles reached a diameter greater than 18mm, final oocyte maturation was triggered by administering Triptorelin - 0.2 mg was administered at trigger, followed by triptorelin 0.1 mg 12 hours later. This protocol was used universally for all patients. Human chorionic gonadotropin (HCG) was only administered if the estradiol (E2) levels were below 5000pg/ml. Throughout the treatment cycles, blood samples were collected from all participants on specific days to analyze serum follicle-stimulating hormone (FSH), luteinizing hormone (LH), estradiol, and progesterone levels. These samples were taken on day 2 of the menstrual cycle, on the day of trigger administration, and the day of oocyte pickup (OPU). All participants underwent endometrial preparation for Frozen Embryo Transfer (FET) after controlled ovarian stimulation. The endometrial preparation was done with oral estradiol valerate universally for all patients starting dose 2mg thrice daily for all patients and escalated if required after assessing endometrium on day 7. Two good-grade blastocysts were selected for transfer into the endometrial cavity.

### Outcome variables

The primary outcome measures included the duration of stimulation, total follicle-stimulating hormone (FSH) used, the number of retrieved oocytes, fertilization rate, blastulation rate, FSH per oocyte used, the incidence of premature luteinizing hormone (LH) surge, number of mature MII (metaphase II) oocytes, cycle cancellation rate, embryo utilization rate, viable (good grade) embryos per oocyte retrieved, and the incidence of ovarian hyperstimulation syndrome (OHSS), categorized as mild/moderate or severe/critical. The secondary outcome measures focused on assessing the implantation, ongoing pregnancy, and live birth rates. These measures provided insights into the success of the interventions in terms of embryo implantation, the sustainability of pregnancy, and the ultimate achievement of live births.

### Ethical considerations

This clinical trial was administered per the Declaration of Helsinki and the International Conference on Harmonization-Good Clinical Practice (ICH-GCP) guidelines. The independent ethics committee endorsed the study protocol before any patients were enrolled. Written informed consent was obtained before the patient underwent any protocol-specific screening or research procedures. The research was registered with the Clinical Trials Registry-India (CTRI).

### Data analysis

Sample size power calculation: We conducted a power analysis with 80% power (β=0.20), a significance level (α) of 0.05, and an expected effect size of a 20% difference in proportions (60% *vs*. 40%). The calculation was performed using SAS software.

The data obtained from the study were analyzed using the statistical software R version 4.2.1 (R Core Team, 2022, Vienna, Austria). Descriptive statistics were employed to summarize the continuous parameters, including the mean and standard deviation (SD). Categorical parameters were summarized using frequencies and percentages. Comparison of continuous variables between groups was made by t-test, whereas the chi-square test was employed to compare the categorical variables. A *p*-value of less than 0.05 was considered statistically significant.

## RESULTS

As shown in Table 1, there are no significant differences between the two groups for any of the variables. Age, BMI, hormone levels, and AFC were similar, suggesting that PPOS and CPOS groups had comparable characteristics regarding ovarian function and response to stimulation.

**Table 1:** Characteristics of the study participants.

Variable	PPOS group (n=44)	CPOS group (n=42)	*p* value
Mean age (years)	25.80±2.72	25.50±2.68	0.580
Mean BMI (kg/m^2^)	24.12±3.20	23.60±3.17	0.453
Mean d2/3 FSH (IU/L)	6.34±1.33	6.14±1.49	0.511
Mean d2/3 LH (IU/L)	7.26±4.04	8.71±5.40	0.164
Mean d2/3 E2 (pg/ml)	41.43±13.58	42.72±15.48	0.684
Mean AMH (ng/ml)	8.10±4.00	8.36±3.81	0.756
Mean AFC (number)	20.99±4.27	21.68±4.09	0.454

BMI: Body Mass Index, d2/3 FSH: Mean follicle-stimulating hormone (FSH) on day 2 or 3 (IU/L), d2/3 LH: luteinizing hormone (LH) on day 2 or 3 (IU/L), d2/3 E2: estradiol (E2) on day 2 or 3 (pg/ml), AMH: anti-Müllerian hormone (AMH) (ng/ ml), AFC: Antral Follicular Count (number).

According to Table 2, LH surge occurred in 2.3% (PPOS) and 2.5% (CPOS) with a non-significant *p*-value. Cycle cancellation rates were 9.1% (PPOS) and 10% (CPOS), also non-significant. Birth rates were 57% (PPOS) and 54% (CPOS), with no significant difference. Implantation rates were 45% (PPOS) and 49% (CPOS), also non-significant. Post-trigger LH levels were significantly lower in the PPOS group. There were no reported ovarian hyperstimulation syndrome (OHSS) cases in either group.

**Table 2:** Comparison of outcomes among study participants.

Outcome	PPOS group (n=44)	CPOS group (n=42)	P value
LH Surge	1 (2.3%)	1 (2.5%)	0.9
Cycle cancellation rate	4 (9.1%)	4 (10%)	0.9
Birth Rate	25 (57%)	23 (54%)	0.54
Implantation Rate	19 (45%)	21 (49%)	0.687
Duration of Stimulation	11.30±1.96	11.41±2.02	0.807
Total FSH Used (IU)	2888.95±791.80	2808±834.52	0.655
Retrieved Oocytes	19.58±8.07	21.87±10.02	0.807
MII Oocytes	15.67±6.23	17.08±7.96	0.376
Post-trigger LH (IU/L)	49.68±27.54	71.83±43.43	0.007
OHSS	00	00	NA

## DISCUSSION

In assisted reproductive technologies, ovarian stimulation is a technique used to stimulate the ovaries to produce numerous mature embryos. Controlled ovarian stimulation (COS) is a kind of ovarian stimulation that concentrates on optimizing follicular growth and maturation through hormonal evaluations and medication alterations. These treatments are designed to improve the odds of successful fertilization and embryo creation through procedures such as in vitro fertilization (IVF), giving individuals and couples a greater opportunity to overcome infertility (Sönmezer *et al.*, 2009; Jirge, 2016).

In the present study, there was no significant difference in the occurrence of LH surge between the PPOS group (2.3%) and the CPOS group (2.5%) (*p*=0.9). Chen *et al.* (2019) performed a randomized controlled experiment in poor responders undergoing IVF therapy, comparing progestin (PPOS) to a gonadotropin-releasing hormone (GnRH) antagonist. The research discovered that the incidence of premature LH surge was lower in the PPOS group than in the antagonist group (0% *vs*. 5.88%, *p*<0.05). According to the previous research evaluating PPOS and CPOS in high-responder patients receiving controlled ovarian stimulation, the LH surge rates were comparable in the PPOS and CPOS groups (2.3% *vs*. 2.5%, *p*>0.05) (Gambhir *et al.*, 2022).

In the present study, The PPOS group had a birth rate of 57%, and the CPOS group had a rate of 54%, with no significant difference observed (*p*=0.54). Similarly, in the study by Tu *et al.*, (2022), the live birth rate in the mild stimulation group was 15.3% (40/261), while in the PPOS group, it was 16.9% (15/89), with no significant difference observed (*p*=0.732) (Tu *et al.*, 2022).

In the present study, the PPOS group had an implantation rate of 45%, while the CPOS group had a rate of 49%, with no significant difference found (*p*=0.687). Our findings are in agreement with the previous research in which the FET implantation rate was 20.1% (69/343) in the mild stimulation group and 17.9% (20/112) in the PPOS group, with no significant difference noted (*p*=0.601) (Tu *et al.*, 2022).

Our study found no significant distinction in the duration of stimulation between the PPOS group (11.30±1.96 days) and the CPOS group (11.41±2.02 days), indicating comparable stimulation periods (*p*=0.807). Additionally, the total amount of FSH administered showed similarity, with the PPOS group using 2888.95±791.80IU and the CPOS group 2808±834.52IU, and no statistically significant difference was observed (*p*=0.655). Similar results were reported by Kao *et al.* (2023) in which there was no significant difference in the total gonadotropin dose between the PPOS group (mean 2079IU) and the GnRHant group (mean 2055IU). Similarly, the duration of gonadotropin administration showed no significant distinction, with the PPOS group averaging 9.4 days and the GnRHant group 9.7 days (Kao *et al.*, 2023). Previous research had found there were no statistically significant differences in clinical pregnancy rates (47.8% *vs*. 43.3%), implantation rates (31.9% *vs*. 27.7%), and live-birth rates (42.6% *vs*. 35.5%) between the progesterone group and the control group. These findings indicate a comparable treatment profile between the two groups, highlighting the feasibility of both approaches (Kuang *et al.*, 2015).

In our study, the PPOS group yielded a mean of 19.58±8.07 retrieved oocytes, while the CPOS group had a mean of 21.87±10.02, demonstrating no significant distinction (*p*=0.807). Moreover, there was no significant variance in the number of mature (MII) oocytes between the PPOS group (15.67±6.23) and the CPOS group (17.08±7.96) (*p*=0.376). Similarly, in the research conducted by Kao *et al.* (2023), the oocyte retrieval rate, as determined by the number of retrieved oocytes, exhibited no noteworthy difference, with rates of 88.9% (184/207) for the PPOS group and 88.0% (980/1114) for the GnRHant group (*p*=0.711).

In the present study, the PPOS group had a cycle cancellation rate of 9.1%, while the CPOS group had a rate of 10%, showing no significant difference (*p*=0.9). Similar results were shown by previous studies (Gambhir *et al.*, 2022; Tu *et al.*, 2022).

In the present study, the post-trigger LH levels were significantly lower in the PPOS group (49.68±27.54IU/L) compared to the CPOS group (71.83±43.43IU/L) (*p*=0.007). It was observed in the past study that individuals undergoing ovarian stimulation using the Progesterone-Primed Ovarian Stimulation (PPOS) protocol exhibited a significantly lower incidence of premature luteinizing hormone (LH) surges, along with lower LH levels on the trigger day, in comparison to those subjected to milder ovarian stimulation (Tu *et al.*, 2022).

It is established that controlled ovarian stimulation (COS), which entails the development of multiple ovarian follicles, often results in heightened estrogen production. Elevated estrogen levels can trigger an abrupt LH surge, potentially causing spontaneous ovulation before the scheduled oocyte retrieval. It was found that medroxyprogesterone acetate (MPA) effectively prevented premature LH surges during controlled ovarian hyperstimulation (COH). Despite administering higher doses of human menopausal gonadotropin (hMG), the number of retrieved oocytes in the MPA-treated group was comparable to the control group. LH suppression persisted during ovarian stimulation in the MPA group, with a low incidence of premature LH surges (0.7%) (Kuang *et al.*, 2015; Huang *et al.*, 2021). These findings suggest that MPA can be a valuable oral option for preventing LH surges in COH among high responders.

The mechanism underlying the effectiveness of PPOS in LH surge suppression primarily hinges upon progesterone’s action on the hypothalamus, which contrasts with GnRH analogues, which directly impact pituitary GnRH receptors to induce pituitary downregulation. Progesterone administration during the early follicular phase, prior to estrogen priming, can impede the transmission of estradiol-induced signals via interneuronal pathways connecting estradiol-responsive neurons with GnRH neurons. Furthermore, high progesterone concentrations reduce the frequency of GnRH pulsations, thereby inhibiting LH synthesis and LH surge occurrence (Wang *et al.*, 2016; Massin, 2017). Nevertheless, many facets of the endogenous LH surge and the precise role of progesterone in its modulation remain elusive, necessitating further investigative endeavours to illuminate these processes comprehensively.

## CONCLUSION

Ovarian stimulation with clomiphene or Progesterone offers comparable outcomes (LH surge, cycle cancellation rates, birth rates, implantation rates, duration of stimulation, total FSH used, retrieved and mature oocytes) in high-responders individuals. However, post-trigger LH levels were significantly lower in the PPOS group. Both groups had no reported cases of ovarian hyperstimulation syndrome (OHSS).

### Limitation

The RCT was performed on a small sample size, and these agents’ role in poor/normal responders was not evaluated. The neonatal outcome has not been evaluated.
